# Twice as Nice: The Duff Formylation of Umbelliferone Revised

**DOI:** 10.3390/molecules26247482

**Published:** 2021-12-10

**Authors:** Vladislav V. Skarga, Vadim V. Negrebetsky, Yuri I. Baukov, Mikhail V. Malakhov

**Affiliations:** Institute of Translational Medicine, Pirogov Russian National Research Medical University, 117997 Moscow, Russia; skargavlad@gmail.com (V.V.S.); nmr_rsmu@yahoo.com (V.V.N.); baukov_yui@yahoo.com (Y.I.B.)

**Keywords:** formylation, the Duff reaction, coumarins, umbelliferones, 8-formylumbelliferone, 6-formylumbelliferone

## Abstract

More efficient and preferably more convenient and greener synthetic solutions in coumarin scaffold functionalization are in steady demand. The Duff *ortho*-formylation of unsubstituted umbelliferone was revised in this study. The reaction conditions were optimized based upon data from the literature analysis and resulted in unexpectedly rapid *ortho*-formylation of umbelliferone, yielding a mixture of *ortho*-formyl position isomers. Thorough studies on the separation of *ortho*-formylated umbelliferones using chromatographic and recrystallization methods as well as the evaluation of their solubility in common organic solvents led to complete resolution of 8-formyl- and 6-formylumbelliferones. The precise protocol for simultaneous preparation, extraction, and purification of 8-formyl- and 6-formylumbelliferones is provided, and the prospective studies of biological and pharmacological activities of these compounds are synopsized.

## 1. Introduction

Coumarins are a large family of natural and synthetic compounds with a variety of biological activities and bioanalytical applications [1,2,3,4,5]. Thus, novel approaches in coumarin scaffold functionalization are in steady demand, and the known strategies are permanently needed in more efficient and preferably more convenient and greener synthetic solutions, therefore assigning a large and still growing number of research publications in this field [6,7].

The Duff reaction is an example of regioselective derivatization routinely used for *ortho*-formylation of aromatic phenols [8]. In turn, umbelliferones (7-hydroxycoumarins) are among the most abundant, widely available, and applicable coumarins [1,2,3,4,5]. The Duff *ortho*-formylation of umbelliferones was numerously reported earlier [9,10,11,12,13,14,15,16,17,18,19], while the vast majority of reports were notably focused on the preparation of 8-formylumbelliferones, which are well-described and routinely used dyes. With that, the data on the production of the 6-formylumbelliferones during the Duff reaction was nearly absent in literature [16,18]. This was found to be rather surprising, since according to the theoretical DFT study on the Duff reaction regioselectivity for non-symmetrically substituted phenols [20] we might suggest the simultaneous formation of 6-formyl and 8-formyl substituted umbelliferones as a result of the Duff *ortho*-formylation, albeit the latter being the predominant product.

Considering the rising trend in the studies of 6-formylumbelliferone (6-FUmb) biological activities [21,22,23,24], its prospective beneficial spectral properties based on the relevance of 6-formyl vs. 8-formyl positioning [4,25], and the routine usage of 8-formylumbelliferone (8-FUmb) as a reference compound for the above-mentioned studies, we found it important to revise the Duff *ortho*-formylation of unsubstituted umbelliferone (Umb) and re-evaluate the 8-FUmb and 6-FUmb yields in this convenient and cost-effective reaction.

## 2. Results

### 2.1. Optimization of the Duff Reaction Conditions

Based upon data from the literature analysis concerning the methodology of the Duff *ortho*-formylation of umbelliferones [9,10,11,12,13,14,15,16,17,18,19], we could draw two relevant conclusions. Firstly, the vast majority of the published information was aimed on the synthesis of 8-FUmb and/or its substituted analogues [9,10,11,12,13,14,15,17,19], whereas the production of 6-FUmb was poorly, if at all, represented [16,18]. Secondly, while the general methodology of the Duff reaction remained the same through the years, the actual protocols of its performance varied significantly, whether it pertains to the reaction conditions (reaction time, reaction temperature, selection of a catalyzing acid, etc.) or to the quantitative ratio of starting umbelliferones and hexamethylenetetramine (HMTA).

Recently, the microwave-assisted modification of the Duff reaction was described [14,16]. Relative to 4-methylumbelliferone, this sufficient modification enabled a notable increase in the yields of *ortho*-formylated products from ~25% (an average yield under common conditions) to 40% (under 300 W microwave treatment for 7 min in AcOH) [14] or even 64% (under 800 W microwave treatment for 3 min in TFA) [16]. Unfortunately, this approach was found to not be applicable for unsubstituted Umb *o*-formylation, in the course of which the formation of yellow-red resin was observed. This process was visually similar to that observed earlier for the psoralen ozonolysis procedure also aimed at the preparation of 6-FUmb [26], and it was presumed that both microwave treatment and ozonolysis might lead to a polymerization of starting material (Umb) and/or its formylated products.

Our brief analysis of the literature data was summarized in Table 1, from which some useful indications could be found.

**Scheme 1:**
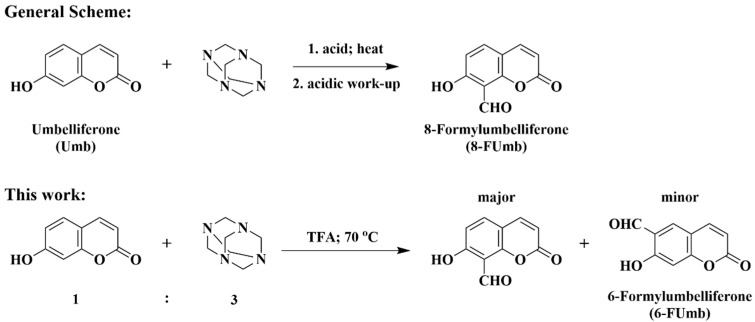
The methodology of the umbelliferone *ortho*-formylation in the Duff reaction.

Firstly, the synthetic experiments described in Table 1 were predominantly focused on the Duff formylation of 4-methylumbelliferone using a HMTA:starting material ratio equal of close to 2:1~3:1, with the latter ratio giving higher yields. Secondly, the higher reaction yields were also reached when TFA was used instead of commonly used acetic acid. These observations allowed us to design a reaction condition, as outlined below in the Scheme 1. These conditions comprised the reacting of Umb and HMTA at 1:3 ratio in TFA at 70 °C. The chosen reaction temperature was close but did not exceed the boiling temperature of TFA (~72 °C) in an attempt to avoid the undesired polymerization.

### 2.2. Ortho-Formylation of Umbelliferone in the Duff Reaction under Chosen Reaction Conditions

**Figure 1:**
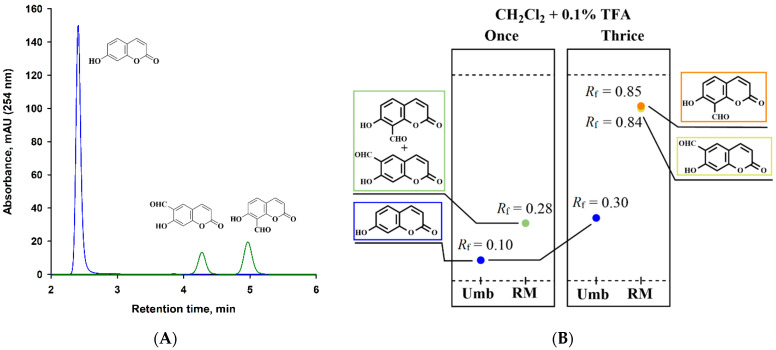
Detection of *ortho*-formylated umbelliferones as the products of the Duff reaction: (**A**) HPLC chromatogram of the reaction mixture before (blue) and after (green) 30 min reaction under the chosen reaction conditions. Conditions: column: Agilent Zorbax^®^ Eclipse XDB C18 Solvent Saver Plus (3.5 μm; 3.0 × 75 mm); mobile phases: (**A**) water + 0.01% TFA, (**B**) acetonitrile + 0.01% TFA; gradient: 80% A and 20% B for 10 min; flow rate: 0.6 mL min^−1^; temperature: 30 °C; injection volume: 20 μL; DAD detection: 254 nm; (**B**) Schematized representation of TLC analysis of the starting Umb and the reaction mixture (RM) after reacting for 30 min. Conditions: TLC Plates: Merck “TLC Silica gel 60” single (**left panel**) or triple (**right panel**) elution with CH_2_Cl_2_ + 0.1% TFA; visualization: 365 nm.

The Duff reaction was performed under the chosen conditions, and our first check at the timepoint of 30 min surprisingly demonstrated that the reaction was already complete (Figure 1A). HPLC analysis of the reaction mixture revealed the full consumption of starting Umb (peak with Rt = 2.407 min) along with an appearance of two product peaks with Rt = 4.274 min and Rt = 4.981 min. The absorption spectrum of the first eluting product with Rt = 4.274 min corresponded to a standard of 6-FUmb previously synthesized according to Chen et al. [27]. The absorption spectrum of the second eluting product with Rt = 4.981 min was similar to that of 8-FUmb, previously described in literature [28]. A further increase in reaction time up to 4 h had no sufficient effect (Figure S1).

Thus, the unexpectedly rapid *ortho*-formylation of Umb in the Duff reaction yielding a mixture of *ortho*-formyl position isomers was clearly demonstrated.

Moreover, it was found that the common stage of the acidic work-up of the reaction mixture was not necessary in our conditions. This final procedure serves for the hydrolysis of intermediate Schiff bases, which are formed in the course of the Duff reaction. In our case, the presence of TFA in the reaction mixture seemed to be enough for reaching the Schiff base hydrolysis in situ. HPLC analysis of the reaction mixture before and after the acidic work-up with 1 M aq. HCl solution found no beneficial effects on the yield of the products (Figure S2). According to HPLC data, a combined yield of 8-FUmb and 6-FUmb in the reaction was estimated equal to 71%, and this yield was higher than those earlier described in literature (see Table 1 for examples and references).

TLC analysis with UV-A visualization (365 nm) spotted the starting Umb with *R*_f_ = 0.10 (blue fluorescence) and the final reaction mixture (after reacting for 30 min) with *R*_f_ = 0.28 (pale yellow-green fluorescence), with the latter presumably comprising both *ortho*-formylated umbelliferones (Figure 1B, left panel). TLC analysis performed in triplicate led to a poor spot separation with an appearance of two spots with *R*_f_ = 0.84 (pale yellow fluorescence) and *R*_f_ = 0.85 (orange fluorescence) (Figure 1B, right panel), which were further attributed to 6-FUmb and 8-FUmb, respectively. Similarly, the normal phase chromatography under the same elution conditions resulted in a very limited (~5%) isolation of pure 8-FUmb, while a majority of 8-FUmb eluted in admixture with 6-FUmb. Therefore, these separation conditions were recognized as inefficient, and a detailed study on solubility-based separation of two *ortho*-formylated umbelliferones was further performed.

### 2.3. Solubility-Based Separation and Characterization of Ortho-Formylated Umbelliferones

Prior to solubility testing, a reaction mixture containing 8-FUmb and 6-FUmb was cooled to room temperature and diluted with methylene chloride (50 mL) acidified with TFA (0.1%) and saturated brine (50 mL) acidified with a 1 M aq. HCl solution to pH 2 to fully protonate 7-OH group of both *ortho*-formylated umbelliferones and enhance the extraction recovery. Then, the resulted mixture was extracted with CH_2_Cl_2_ (3 × 50 mL) acidified with TFA (0.1%). The combined organic extracts were washed with saturated brine (3 × 50 mL) acidified with a 1 M aq. HCl solution (pH = 2), dried over Na_2_SO_4_, filtered, concentrated to dryness using rotary vacuum evaporator, and tested for solubility in a variety of common organic solvents (Table 2).

The results of the solubility test indicated the limited solubility of 8-FUmb and 6-FUmb mixture in the most common HPLC solvents (e.g., acetonitrile or methanol). Thus, it became obvious that HPLC could be used for analytical purposes only and not for the purpose of (semi-)preparative isolation/purification.

Recrystallization was suggested as a useful method for separation of 8-FUmb and 6-FUmb, although earlier it was reported only for 8-FUmb [12,13,16,19], but not for the mixtures of 8-FUmb and 6-FUmb. We have tested this hypothesis using all the solvents in the solubility range from “poor” to “good” according to Table 2. It was found that recrystallization from ethanol, isopropanol, and acetonitrile effectively resulted in precipitation, while the FUmb composition of the precipitated solid was analogous to that of the FUmb mixture in solution. This might be due to the equally limited solubility for both FUmbs in these solvents. Hot methanol was found to be the only solvent which allowed for the effective isolation of the target compound by means of recrystallization: the single recrystallization afforded needles of 8-FUmb with a purity of 98% (with 2% impurity of 6-FUmb), which could be repeatedly raised up to 99.99% after 5 cycles of recrystallization. Unfortunately, this approach made it possible to isolate only ~30% of 8-FUmb, whereas the remaining 70% of 8-FUmb were still in admixture with 6-FUmb in the methanolic solution. Isolation of ~30% of 8-FUmb as a pure crystal solid corresponded to an overall yield of ~21%, which in turn could be considered as a reasonable result according to literature data, but not acceptive for the purposes of the present study.

A good solubility in chloroform and methylene chloride suggested the applicability of these solvents as an eluent for normal phase silica gel column chromatography. Series of screening experiments finally resulted in the selection of a four-component solvent system comprising of methylene chloride/petroleum ether/ethyl acetate/acetic acid in the ratio of 45/45/10/1, which provided complete resolution of 8-FUmb and 6-FUmb during preparative normal phase silica gel column chromatography with the final yields of 39% and 6.8% for 8-FUmb and 6-FUmb, respectively.

Both of the isolated *ortho*-formylated umbelliferones were of high-purity grade and further characterized using ^1^H and ^13^C Attached Proton Test (APT) NMR spectroscopy as well as high resolution mass-spectrometry (Figures S3–S8).

The precise protocol for preparation, extraction, and purification of 8-FUmb and 6-FUmb is outlined below in the Materials and Methods section.

### 2.4. Photophysical Properties of 8-FUmb and 6-FUmb

**Figure 2:**
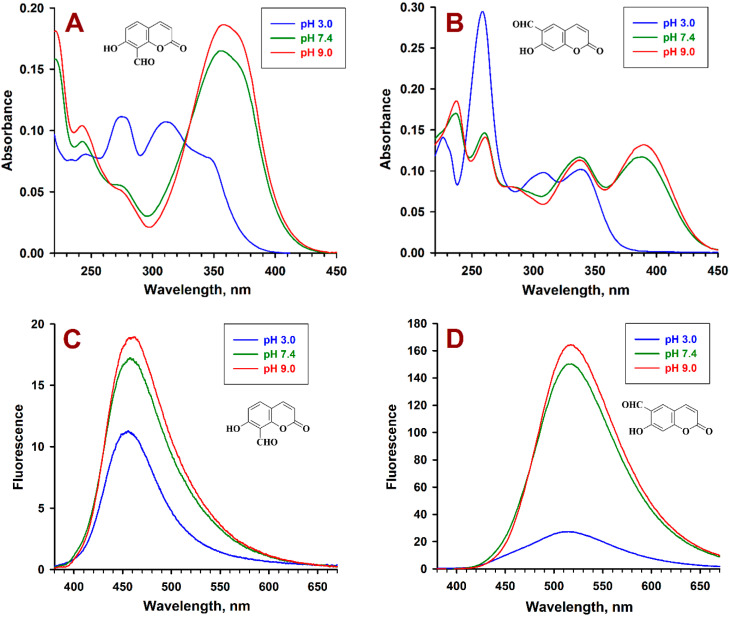
Absorbance spectra of 8-FUmb (**A**) and 6-FUmb (**B**) at various pH, and corresponding fluorescence spectra of 8-FUmb (**C**) and 6-FUmb (**D**) under excitation at 347 nm. Conditions: pH 3.0 = aqueous solutions of 8-FUmb and 6-FUmb were acidified with 1 M aq. HCl to the indicated pH value; pH 7.4 = solutions of 8-FUmb and 6-FUmb in phosphate buffered saline (PBS); pH 9.0 = aqueous solutions of 8-FUmb and 6-FUmb were basified with 1 M aq. NaOH to the indicated pH value. Concentration: 10 µM, 1% of ethanol.

Further, the photophysical properties of isolated and purified 8-FUmb and 6-FUmb were assessed. UV-vis absorption and fluorescence emission spectra were recorded for both *ortho*-formylated umbelliferones (Figure 2). The spectral studies were performed in aqueous phase under acidic (pH 3.0), neutral (pH 7.4), and mild alkaline (pH 9.0) conditions. The chosen acidic pH value corresponded to a pH range where 7-OH groups of both 8-FUmb and 6-FUmb were fully protonated. The alkaline conditions were mild enough to deprotonate the 7-OH groups affording anionic form of both umbelliferones, but not too alkaline to induce a solvolytic pyrone ring opening [29].

UV-vis absorption spectrum of 8-FUmb at pH 3.0 was characterized with three absorption maxima at 245, 275, and 310 nm with a vivid schedule near 350 nm and no absorption in the visible region (Figure 2A). An increase in pH values (to neutral and alkaline region) was accompanied with the dramatic changes of the absorption spectra, which may be attributed to a dissociation of the 7-OH protons [30]. At both pH 7.4 and pH 9.0, the maxima at 245 and 275 nm were transformed to a barely visible shoulder at ~275 nm, and a new broad maximum at 355 nm appeared, as well as an absorption in the visible region. The close absorbances at pH 7.4 and pH 9.0 might indicate an almost complete deprotonation of 8-FUmb at physiological conditions. The similar pH-dependent changes in UV-vis absorption spectra of 6-FUmb were also detected (Figure 2B). Both at pH 7.4 and pH 9.0, the characteristic maximum at 259 nm reduced by half. More notably, a new absorption maximum was bathochromically shifted (388 nm for 6-FUmb vs. 355 nm for 8-FUmb), and the absorption in the visible region was more prominent.

Fluorescence of both *ortho*-formylated umbelliferones was also pH-dependent (Figure 2C,D). The fluorescence emission spectra of 8-FUmb at pH 3.0 had a maximum at 455 nm (Figure 2C). Increasing the pH value resulted in a slight bathochromic shift of the emission maximum (to 457 nm at pH 7.4 and 461 nm at pH 9.0) along with ~2-fold increase in fluorescence intensity. These spectral features are in good agreement with the data on weak blue fluorescence of 8-FUmb previously described [28]. The broad fluorescence emission spectra of 6-FUmb was shifted to green spectral region and had a maximum faintly dependent on pH value (515 nm at pH 3.0 and 517 nm at pH 7.4 and pH 9.0) (Figure 2D). More importantly, the fluorescence intensities of 6-FUmb were notably higher than those of 8-FUmb. The fluorescence of protonated 6-FUmb at pH 3.0 was about twice higher relative to 8-FUmb and about nine times higher at pH 7.4 and pH 9.0.

The fluorescence quantum yields (*Φ*) for 8-FUmb and 6-FUmb at various pH were measured relative to quinine sulfate in 0.5 M H_2_SO_4_ (*Φ* = 0.546) used as a standard (Figure S9). The same calculations were also made for Umb, a widely used fluorophore with a well-known pH dependence of fluorescence [30]. The calculated data confirmed both of our predictions, which were made in the course of spectral studies. Firstly, and unsurprisingly, the higher *Φ* values were found for anionic forms of both FUmbs. Along with spectral data, this finding argued for the complex character of FUmb solutions at neutral pH region, which presumably contain an anionic form of FUmb with a slight admixture of protonated form. Secondly, and more importantly, 6-FUmb was found to be a more effective fluorophore as compared to 8-FUmb at all the pH values studied: the *Φ* values for anionic form of 6-FUmb at pH 7.4 and pH 9.0 were about an order of magnitude higher than those of 8-FUmb (0.093 vs. 0.008 at pH 7.4 and 0.103 vs. 0.009 at pH 9.0, respectively).

pH-dependent photophysical characteristics for all the umbelliferones studied in this work were summarized below in Table 3.

## 3. Discussion

Among the multiple types of biological and pharmacological activities, the therapeutic effects natural and synthetic coumarins on CNS could be stressed [31,32]. Due to their targeted action on the CNS receptors and enzymes, coumarins could be construed as the potential therapeutics for psychiatric and neurodegenerative disorders (e.g., Alzheimer’s and Parkinson’s diseases, schizophrenia, anxiety, epilepsy, and depression).

Using kinetics and molecular docking studies, it was shown that both 6-FUmb and 8-FUmb possessed an anti-Alzheimer’s disease activity via noncompetitive inhibition of BACE1 in vitro, and the activity of 6-FUmb was more pronounced [21]. In addition, 6-FUmb was characterized as a potent inhibitor of ONOO^−^-mediated nitration of tyrosine, being one of the important therapeutic targets in Alzheimer’s disease. Here, it should be expressly pointed out that for the purposes of this and following studies, 6-FUmb was isolated from *Angelica decursiva*, a traditional medicinal plant widely used in Korea, China, and Japan.

Furthermore, the prospective neuroprotective activity of the 6-FUmb cycloadducts with biologically abundant aminothiols (cysteine and homocysteine) was predicted using chemoinformatic analysis [24]. It is known that an elevated level and/or accumulation of homocysteine may result in a large number of neurological conditions (e.g., Alzheimer’s disease, senile dementia, vascular disorders, nephropathy, etc.) [33]. Therefore, the predicted therapeutic activity of the 6-FUmb cycloadducts with homocysteine is of greater interest and may determine the prospects of their therapeutic applicability for the management of the above-mentioned conditions.

The marked anti-inflammatory effects were earlier described for both 6-FUmb and 8-FUmb. 6-FUmb was found to inhibit the over-activated inflammation in LPS-stimulated RAW 264.7 macrophages downregulating the production of NO and pro-inflammatory cytokines via including inflammation-associated signaling pathways, including ERK1/2 and NF-κB [22]. Both 6-FUmb and 8-FUmb, as well as parent Umb, exerted neuroprotective effects by inhibiting monoamine oxidase A (MAO-A), self-amyloid β aggregation, and lipid peroxidation, and could be considered as the multitarget-directed ligands for the treatment of depression [23]. Among the umbelliferones studied in this work, 6-FUmb was characterized as the most potent *h*MAO-A and *h*MAO-B inhibitor, and it was expressly signed that the formyl substituent of the umbelliferone scaffold plays a crucial role in the inhibition of MAOs as compared to hydroxyl, methoxyl, and carboxyl groups. Also, the fluorogenic umbelliferone derivative exhibited high affinity to macrophage migration inhibitory factor (MIF) tautomerase, which was described as a regulator of the enzymatic activity, playing key roles in inflammation and cancer [34].

Synthesis of 6-FUmb allows for further research of its role in specific immunosuppression, which was described for 6-FUmb, containing a crude mixture of psoralen photooxidation products in our previous studies [35,36,37]. In tests on transformed T lymphocytes of Jurkat cell line, 6-FUmb exhibited an apoptogenic effect by means of the mitochondrial permeability transition pore opening, whereas the viability of normal T lymphocytes from healthy individuals was not affected [18]. This suggests the crucial role of 6-FUmb in therapeutic effects of psoralen photochemotherapy and contemplates resuming the studies of immunotropic activity of 6-FUmb.

In addition, we could hypothesize that the beneficial effects of the bathochromic shift in both absorption and fluorescence spectra of 6-FUmb vs. 8-FUmb might be very useful in a variety of spectral studies analogous to those previously described for 8-FUmb and its derivatives [28,38].

Considering the relevance of the above-mentioned studies and the results described therein, the synthetic method of simultaneous preparation 6-FUmb and 8-FUmb presented herein might be widely used in future studies.

## 4. Materials and Methods

### 4.1. Materials

Umbelliferone (99% purity) was purchased from Acros Organics (NJ, USA), trifluoroacetic acid (HPLC grade) was purchased from Fisher Scientific (Loughborough, UK), and hexamethylenetetramine (≥99% purity) was purchased from Sigma-Aldrich (Sigma-Aldrich, St. Louis, MO, USA). Other reagents and solvents were purchased from commercial suppliers (Sigma-Aldrich, St. Louis, MO, USA; Acros Organics, Geel, Belguim; Fisher Scientific, Loughborough, UK; J.T. Baker, Deventer, Netherlands) and used without additional purification. All the organic solvents were of HPLC grade.

Ultra-pure water (18.2 MΩ cm) produced on an OMNI-Analytic water purification system (Xiamen RSJ Scientific Instruments Co. Xiamen, China) was used for preparation of all aqueous solutions, as well as for spectral and HPLC analyses. Phosphate buffered saline (PBS, pH 7.4 ± 0,1, Biotechnology grade, VWR Life Science AMRESCO, Solon, OH, USA) was prepared by dissolving the tableted PBS in ultra-pure water. Stock solutions of all the umbelliferones were prepared in distilled ethanol and stored in the dark.

### 4.2. Analytical Procedures

All analytical procedures were performed on air at ambient temperature (23 ± 2 °C).

UV-vis absorption spectra were recorded in a 1.0 cm path length quartz cuvette (Hellma Analytics, Müllheim, Germany) using a Shimadzu UV-1601PC spectrophotometer.

The corrected fluorescence emission spectra were recorded in a 1.0 cm path length quartz cuvette (Hellma Analytics, Müllheim, Germany) using a Hitachi F-7000 spectrofluorometer. Both excitation and emission slits were set at 5 nm. To minimize the reabsorption effect, fluorescence measurements were performed in samples with absorbances, not exceeding 0.1 at and above the excitation wavelength. The fluorescence quantum yields (*Φ*) for all the umbelliferones were measured relative to quinine sulfate in 0.5 M H_2_SO_4_ (*Φ* = 0.546) as a standard [39].

Analytical reverse phase HPLC analyses were performed using an Agilent 1200 DAD-HPLC system (Agilent Technologies, Santa Clara, CA, USA) in gradient elution mode under conditions indicated in the corresponding figure captions. Data acquisition and processing were made using a ChemStation^®^ software system (Agilent Technologies, Waldbronn, Germany).

^1^H NMR and ^13^C APT NMR spectra for both *ortho*-formylated umbelliferones were recorded on Bruker DPX 300 FT-NMR spectrometer (at 300 MHz and 75 MHz, respectively) using tetramethylsilane as an internal standard and DMSO-*d*_6_ as a solvent.

The mass spectra for both *ortho*-formylated umbelliferones were recorded in positive mode using an Orbitrap Elite mass-spectrometer (Thermo Fisher Scientific, Waltham, MA, USA) equipped with a HESI probe (direct infusion rate = 5 μL min^−1^; spray voltage = 4 kV).

The pH values were measured using a Hanna HI 83141 pH meter and corrected by addition of 1 M aq. HCl or 1 M aq. NaOH, as appropriate.

### 4.3. Preparation and Characterization of Ortho-Formylated Umbelliferones

In a 50 mL round-bottom flask, umbelliferone (324 mg, 2 mmol) and hexamethylenetetramine (841 mg, 6 mmol) were dissolved in 20 mL of TFA. The reaction mixture was heated to 70 °C, stirred at this temperature for 30 min, cooled to room temperature, and diluted with CH_2_Cl_2_ (50 mL) acidified with TFA (0.1%) and saturated brine (50 mL) acidified with a 1 M aq. HCl solution (pH = 2). Then, the mixture was extracted with CH_2_Cl_2_ (3 × 50 mL) acidified with TFA (0.1%). The combined organic extracts were washed with saturated brine (3 × 50 mL) acidified with a 1 M aq. HCl solution (pH = 2), dried over Na_2_SO_4_, filtered, and concentrated to dryness using rotary vacuum evaporator. The resulting dry residue (271 mg) was purified by normal phase column chromatography on silica gel (Kieselgel 60, 40–63 μm, Merck) eluting with CH_2_Cl_2_/ethyl acetate/petroleum ether/AcOH (45/45/10/1) to yield 8-formylumbelliferone (148 mg, 0.779 mmol, 39%) as an off-white solid and 6-formylumbelliferone (26 mg, 0.137 mmol, 6.8%) as a pale-yellow solid.

Characterization of *ortho*-formylated umbelliferones:

8-formylumbelliferone: ^1^H-NMR (300 MHz, DMSO-*d*_6_, ppm): δ 6.37 (d, *J* = 9.6 Hz, 1H), 6.94 (dd, *J* = 8.8, 0.5 Hz, 1H), 7.86 (d, *J* = 8.8 Hz, 1H), 8.01 (d, *J* = 9.6 Hz, 1H), 10.41 (d, *J* = 0.4 Hz, 1H), 11.90 (s, 1H). ^13^C APT NMR (75 MHz, DMSO-*d*_6_, ppm): δ 109.20, 111.15, 112.58, 113.95, 136.26, 144.53, 155.69, 159.13, 163.93, 190.84. HRMS (HESI): *m/z* calc. for [M + H]^+^: 191.0339; found: 191.0343.

6-formylumbelliferone: ^1^H-NMR (300 MHz, DMSO-*d*_6_, ppm): δ 6.33 (d, *J* = 9.6 Hz, 1H), 6.87 (s, 1H), 8.06 (s, 1H), 8.07 (d, *J* = 9.6 Hz, 1H), 10.26 (s, 1H), 11.69 (br. s, 1H). ^13^C APT NMR (75 MHz, DMSO-*d*_6_, ppm): δ 103.47, 111.96, 113.16, 120.48, 130.00, 144.48, 158.75, 159.51, 163.41, 189.22. HRMS (HESI): *m/z* calc. for [M + H]^+^: 191.0339; found: 191.0343.

## 5. Conclusions

In our study, unsubstituted umbelliferone *ortho*-formylation conditions in the course of the Duff reaction were optimized based upon data from the literature analysis. This resulted in the simultaneous and unexpectedly rapid formation of the *ortho*-formyl position isomers with a total yield superior to those previously described in literature. Conducting the reaction under an optimized protocol needed no routine stage of acidic work-up, and thorough studies on separation of the *ortho*-formylated umbelliferones resulted in the first-ever precise, convenient, and cost-effective protocol for simultaneous preparation, extraction, and purification of 8-formyl- and 6-formylumbelliferones with a moderate yield. Spectral photophysical studies revealed the distinct fluorescence behavior of these compounds, nearly identical in terms of their chemical characteristics. Considering the rising trend in the studies of 8-formyl- and especially 6-formylumbelliferone biological activities, the optimized protocol for simultaneous preparation of these compounds presented herein might be widely used in future studies.

## Figures and Tables

**Table 1:** Brief summary of the Duff reaction conditions previously described for *ortho*-formylation of umbelliferones.

Starting Material	Reaction Conditions ^1^				Yield (%)	Reference ^2^
	HMTA:SM Ratio	Temperature (°C)	Reaction Time	Solvent		
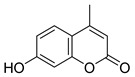	7:3	Boiling	5.5 h	AcOH	10	[9]
	~2:1	Boiling	Overnight	AcOH	14.5	[10]
	2:1	95	5.5 h	AcOH	15.5	[11]
	3:1	Boiling	4-5 h	AcOH	18	[12]
	3:1	80-85	6 h	AcOH	22	[13]
	3:1	Boiling	8 h	AcOH	25	[14]
	3:1	MW 300 W	7 min	AcOH	40	[14]
	~3:2	Boiling	8 h	TFA	57	[15]
	3:1	MW 800 W	3 min	TFA	64	[16]
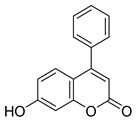	10:1	n. a.	6-8 h	AcOH	60	[17]
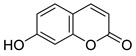	5:3	Boiling	10 min	TFA	n. a.	[18]
	3:1	n. a.	1 h	AcOH	44	[19]

**^1^** HMTA = hexamethylenetetramine; SM = starting material; MW = microwave irradiation treatment; n. a. = not available. **^2^** References to Table 1 are compliant with those presented below in the general References section.

**Table 2:** Solubility of 8-FUmb and 6-FUmb mixture.

Solubility	Solvent	
Excellent (>100 mg/mL)	Dimethylformamide	
	Dimethyl sulfoxide	
Good (10–100 mg/mL)	Methylene chloride	
	Chloroform	
Limited (1–10 mg/mL)	Methanol	Acetonitrile
	Ethanol	Acetic acid
Poor (0.1–1 mg/mL)	Acetone	Isopropanol
	Diethyl ether	
Insoluble (<0.1 mg/mL)	Water	Toluene
	Ethyl acetate	Hexane

**Table 3:** Photophysical characteristics of *ortho*-formylated umbelliferones.

Compound	Parameter ^1^	pH 3.0 ^2^	pH 7.4 ^2^	pH 9.0 ^2^
8-FUmb (pK 6.54) ^3^	Absorption	245 (8100)	243 (9100)	242 (10,400)
		275 (11,100)	275sh (5500)	275sh (5100)
		310 (10,700)		
		350sh (7400)	355 (16,500)	357 (18,600)
	Emission	455 (0.006)	457 (0.008)	461 (0.009)
6-FUmb (pK 6.55) ^3^	Absorption	227 (14,100)	237 (17,000)	238 (18,500)
		259 (29,500)	260 (14,600)	261 (14,100)
		308 (9800)		
		338 (10,200)	338 (11,700)	338 (11,300)
			388 (11,700)	389 (13,200)
	Emission	515 (0.017)	517 (0.093)	517 (0.103)
Umb ^4^	Absorption	324 (21,100)	327 (11,900)	
			366sh (6600)	366 (22,900)
	Emission	457 (0.802)	457 (0.903)	457 (0.918)

^1^ Absorption = peak maxima in nm (molar extinction coefficient in (M cm)^−1^); Emission = peak maximum in nm (fluorescence quantum yield). ^2^ Conditions: pH 3.0 = aqueous solutions acidified with 1 M aq. HCl to the indicated pH value; pH 7.4 = solutions in phosphate buffered saline (PBS); pH 9.0 = aqueous solutions basified with 1 M aq. NaOH to the indicated pH value. Concentration: 10 µM, 1% of ethanol. ^3^ pK values were obtained photometrically (for details see Figure S10). ^4^ As a widely used reference, fluorophore with a well-known pH dependence of fluorescence and a starting material.

## Data Availability

Data are contained within the article and supplementary materials.

## Supplementary Materials

The following are available online. Figures S1–S10 (HPLC data; NMR and HRMS spectra; spectral data for fluorescence quantum yield calculations).

## Sample Availability

Not available.
