# Supplementary material for: Twice as Nice: The Duff Formylation of Umbelliferone Revised
Source: Molecules. 2021 Dec 10;26(24):7482. doi: 10.3390/molecules26247482 (PMC8706561; doi:10.3390/molecules26247482)
Supplement: Supplementary file 1 [file molecules-26-07482-s001.zip › molecules-1476032-supplementary.pdf]

SUPPLEMENTARY MATERIALS

Twice as nice: the Duff formylation of umbelliferone revised

Vladislav V. Skarga [1], Vadim V. Negrebetsky [1], Yuri I. Baukov [1] and Mikhail V. Malakhov [1,*]

[1] Institute of Translational Medicine, Pirogov Russian National Research Medical University, 1 Ostrovityanov str.; Moscow 117997, Russian Federation; skargavlad@gmail.com (V.V.S.); nmr_rsmu@yahoo.com (V.V.N.); baukov_yui@yahoo.com (Y.I.B.)

* Correspondence: malakhov.mikhail@gmail.com; Tel.: +7-916-815-5258

Content

Figure S1 HPLC chromatogram of reaction mixture after reaction for 30 min of 4 hours.

Figure S2 HPLC chromatogram of reaction mixture before and after acidic work-up.

Figure S3 ^{1}H NMR spectrum of 8-formylumbelliferone.

Figure S4 ^{13}C APT NMR spectrum of 8-formylumbelliferone.

Figure S5 ^{1}H NMR spectrum of 6-formylumbelliferone.

Figure S6 ^{13}C APT NMR spectrum of 6-formylumbelliferone.

Figure S7 HRMS spectrum of 8-formylumbelliferone recorded in positive mode.

Figure S8 HRMS spectrum of 6-formylumbelliferone recorded in positive mode.

Figure S9 Fluorescence quantum yield measurements.

Figure S10 pK values for 8-FUmb and 6-FUmb.

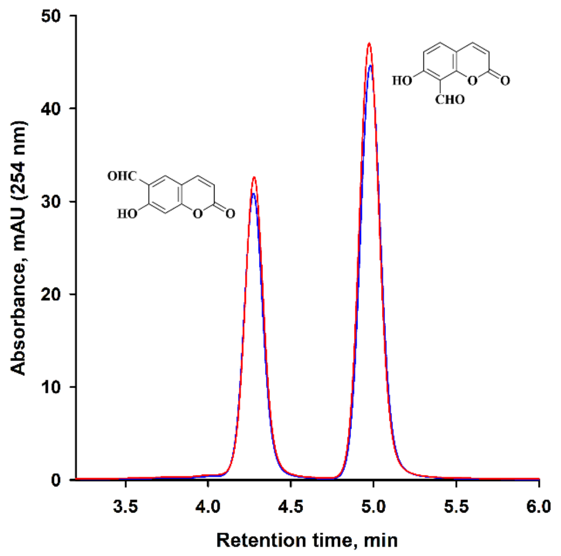

Figure S1 HPLC chromatogram of reaction mixture after reaction for 30 min or for 4 hours.

Colors: blue – reaction for 30 min; red – reaction for 4 hours.

Conditions: column: Agilent Zorbax® Eclipse XDB C18 Solvent Saver Plus (3.5 µm; 3.0 × 75 mm); mobile phases: A – water + 0.01% TFA, B – acetonitrile + 0.01% TFA; gradient: 80% A and 20% B for 10 min; flow rate: 0.6 mL min^{-1}; temperature: 30 °C; injection volume: 20 µL; detection: 254 nm

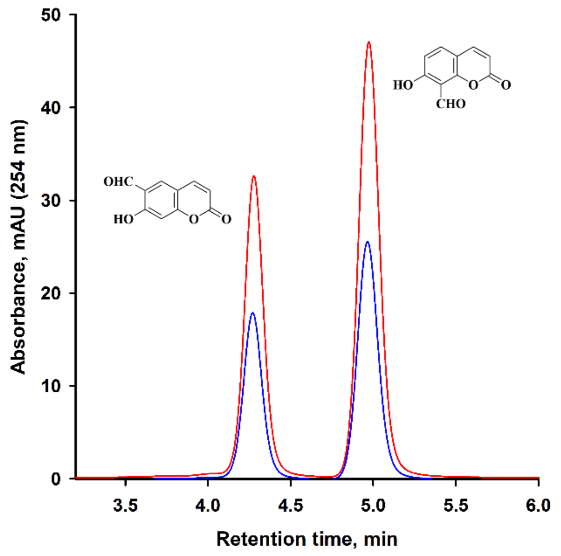

Figure S2 HPLC chromatogram of reaction mixture before and after acidic work-up with 1 M aq. HCl solution.

Colors: red – before acidic work-up; blue – after acidic work-up (2-fold dilution).

Conditions: the same as on Figure S1 presented above.

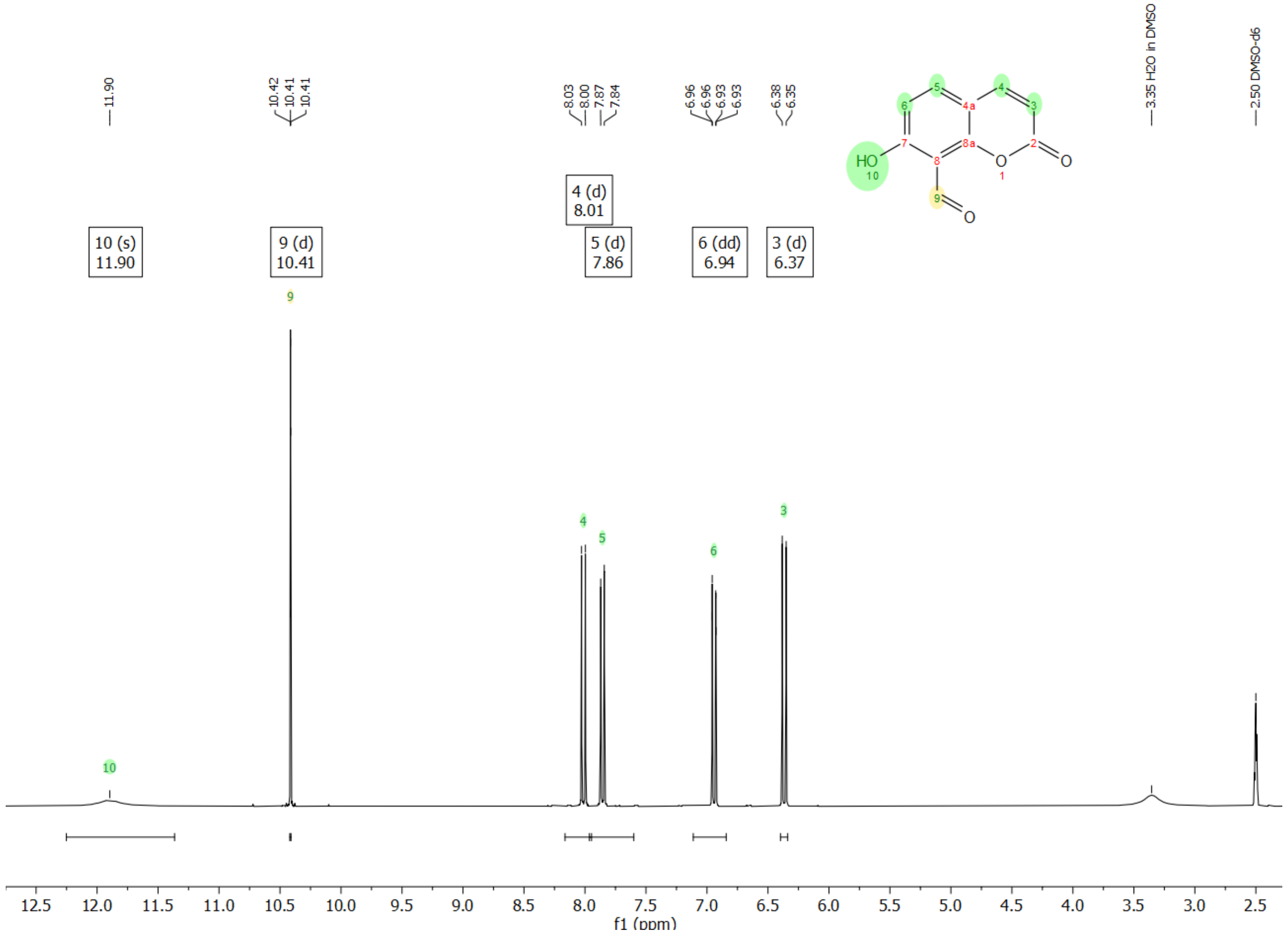

Figure S3 ^{1}H NMR spectrum of 8-formylumbelliferone.

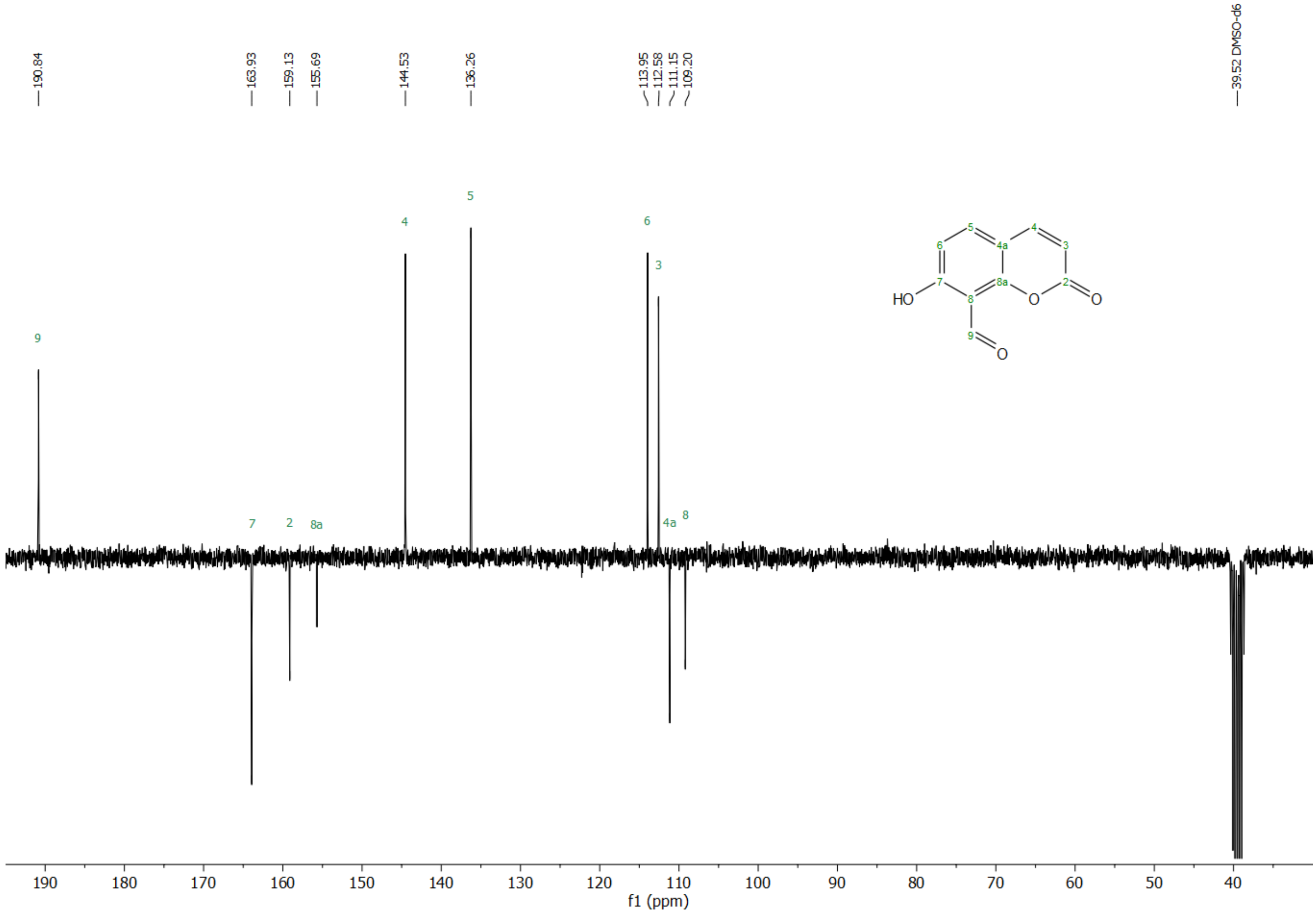

Figure S4 ^{13}C APT NMR spectrum of 8-formylumbelliferone.

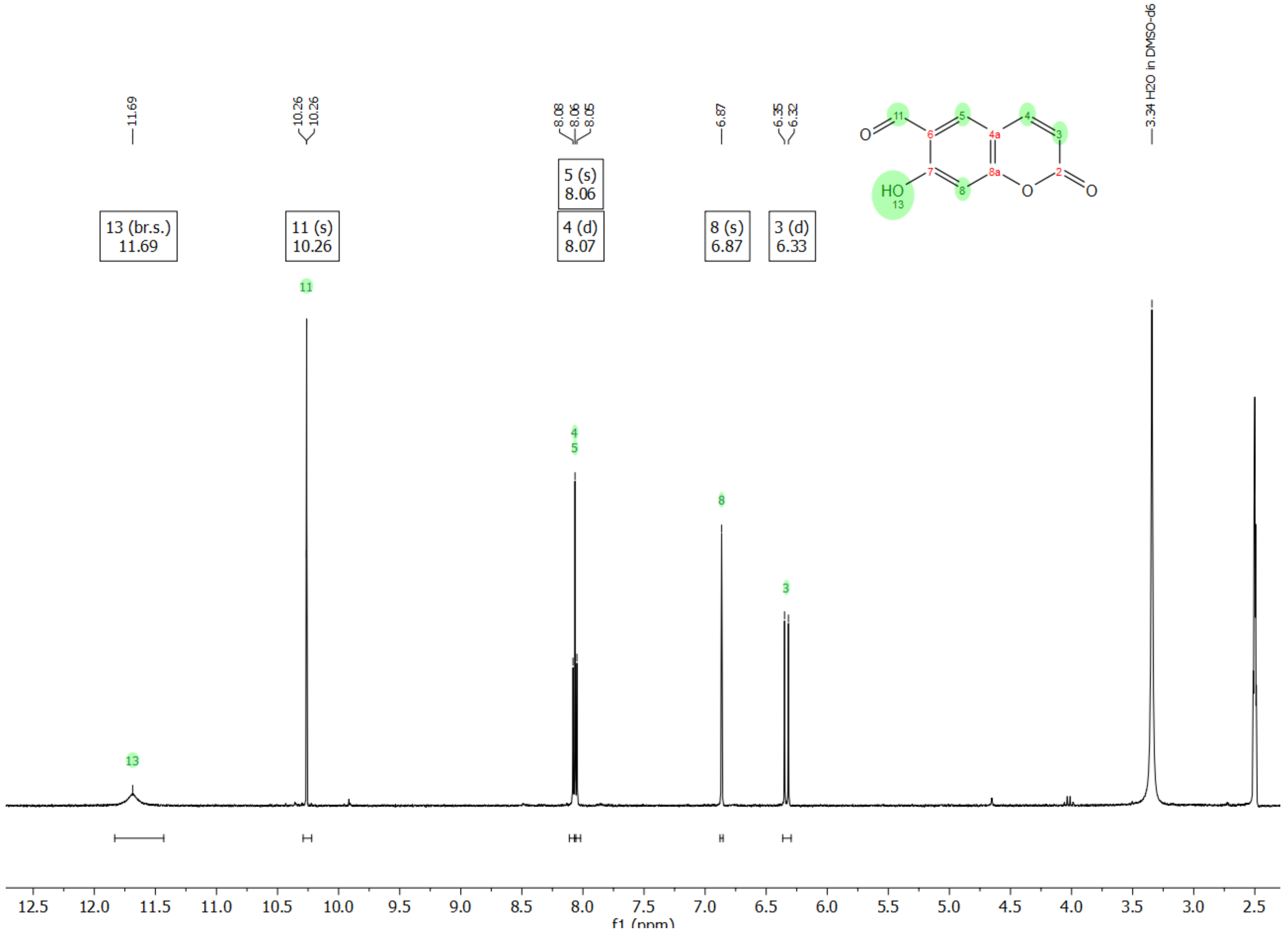

Figure S5 ^{1}H NMR spectrum of 6-formylumbelliferone.

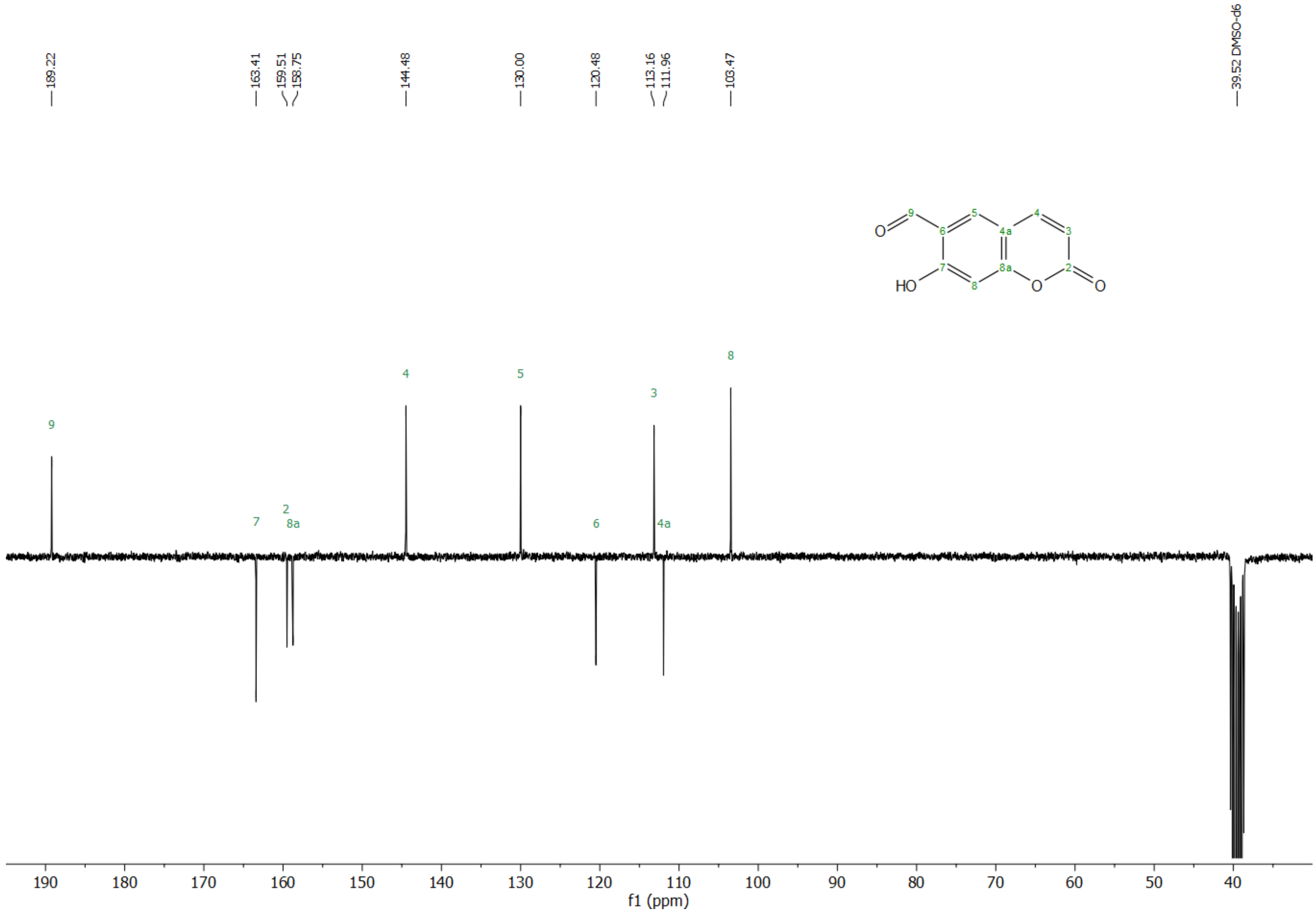

Figure S6 ^{13}C APT NMR spectrum of 6-formylumbelliferone.

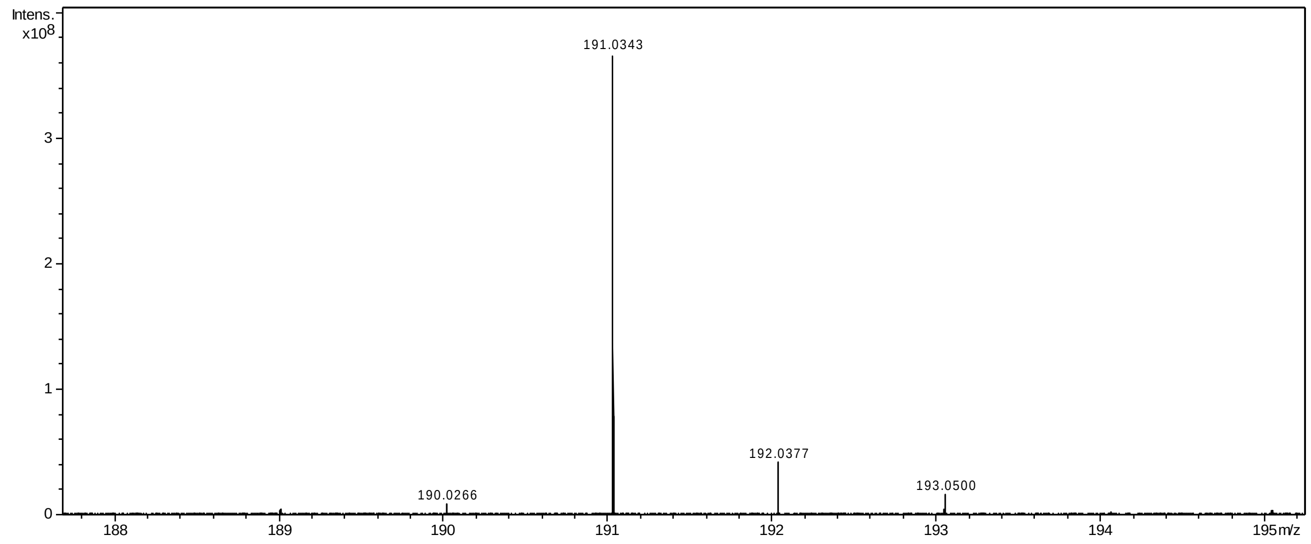

Figure S7 HRMS spectrum of 8-formylumbelliferone recorded in positive mode.

Solvent – acetonitrile/H_2O (50/50) + 0.1% formic acid

HRMS (HESI): *m*/*z* calc. for $[M+H]^+$: 191.0339; found: 191.0343

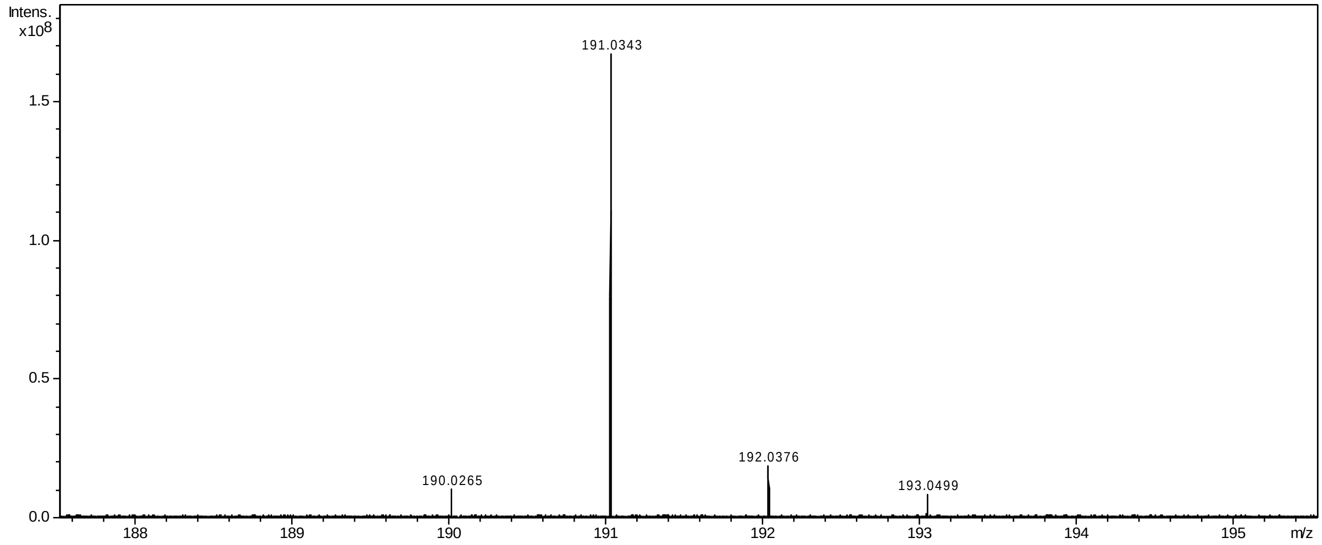

Figure S8 HRMS spectrum of 6-formylumbelliferone recorded in positive mode.

Solvent – acetonitrile/H_2O (50/50) + 0.1% formic acid

HRMS (HESI): *m*/*z* calc. for $[M+H]^+$: 191.0339; found: 191.0343

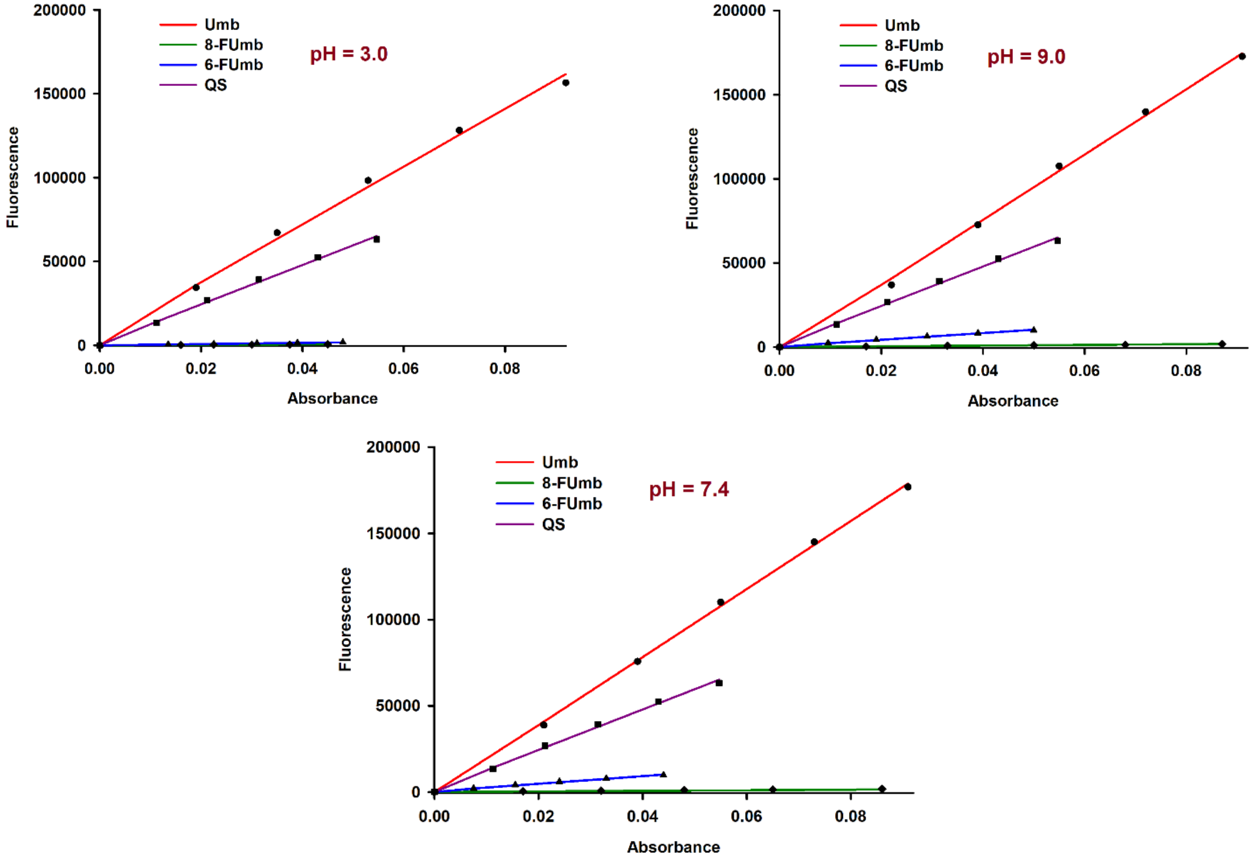

Figure S9 Fluorescence quantum yield measurements.

The relative fluorescence quantum yields for all the umbelliferones at various pH values were calculated using the slope method according to the following equation:

$$\boldsymbol{\Phi}_x = \boldsymbol{\Phi}_{st} \times \frac{\boldsymbol{Slope}_x}{\boldsymbol{Slope}_{st}} \times \frac{\eta_x^2}{\eta_{st}^2}$$

where

$\boldsymbol{\Phi}_x$ is the fluorescence quantum yield of umbelliferone; $\boldsymbol{\Phi}_{st}$ is the fluorescence quantum yield of quinine sulfate in 0.5 M H_2SO_4 ($\boldsymbol{\Phi}_{st}$ = 0.546) used as a standard; $\boldsymbol{Slope}_x$ and $\boldsymbol{Slope}_{st}$ are the integrated fluorescence intensities *vs* absorbance for a series of dilutions of umbelliferones and quinine sulfate, respectively, measured at the same excitation wavelength (437 nm).

To minimize the reabsorption effect, fluorescence measurements were performed in the samples with absorbances not exceeded 0.1 at and above the excitation wavelength.

The refractive indexes were set equal to 1.333 for all the aqueous media.

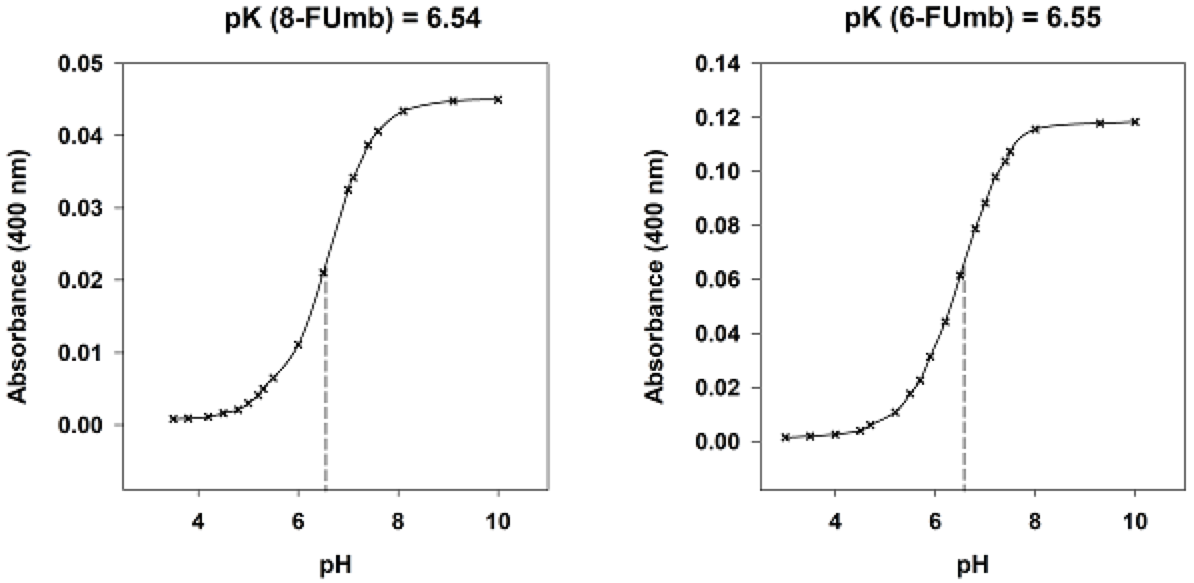

Figure S10 pK values for 8-FUmb and 6-FUmb

pK values for 8-FUmb and 6-FUmb were obtained photometrically by pH titration at 400 nm where protonated forms of both *ortho*-formylated umbelliferones do not absorb (see Figure 2A,B of the article body).

Conditions:

Aqueous solutions of 8-FUmb and 6--FUmb (10 μM, 1% of ethanol) were corrected to the indicated pH values by addition of 1 M aq. HCl or 1 M aq. NaOH as appropriate.

pH values were measured using a Hanna HI 83141 pH meter.
